# Tuberculous Gastric Abscess in a Patient with AIDS: A Rare Presentation

**DOI:** 10.1155/2016/5675036

**Published:** 2016-04-30

**Authors:** Ekta Nayyar, Julian A. Torres, Carlos D. Malvestutto

**Affiliations:** Department of Infectious Diseases and Immunology, New York University School of Medicine, 550 First Avenue, New York, NY 10016, USA

## Abstract

Tuberculosis is a healthcare concern that affects millions of individuals around the globe. Coinfection with HIV has changed both the clinical presentation and the outcome of the disease dramatically in the last few decades. Extrapulmonary tuberculosis is seen more frequently in the immunocompromised host. An unusual case of gastric tuberculosis in an AIDS patient is reported here. A 49-year-old female with AIDS was admitted for fever and epigastric pain. A gastric submucosal abscess was observed on imaging and confirmed by biopsy showing numerous neutrophils and acid-fast bacilli. Aspirate grew* Mycobacterium tuberculosis.* This report highlights a very unusual presentation of tuberculosis in an immunodeficient patient. High clinical suspicion for opportunistic infections in unusual locations should be maintained in these patients presenting with clinical syndromes that do not respond to standard treatments. New diagnostic modalities facilitate accurate identification of these infections.

## 1. Introduction

It is estimated that one-third of the world's population is infected with* Mycobacterium tuberculosis* (MTB) [1]. In 2014, there were 9.6 million new cases and 12% of them were coinfected with HIV [2]. The tuberculosis (TB) case fatality rate is disproportionately elevated among HIV-infected individuals compared to that of those without HIV infection. The annual incidence of TB-HIV coinfection in the US has been declining steadily since the mid-1990s. This trend has been driven, in part, by the improvements in HIV treatment since the introduction of protease inhibitors and the subsequent immunological recovery observed in virologically suppressed HIV-infected patients [3–5].

In most instances, MTB remains latent and controlled by host defenses, but conditions that impair cellular immunity, like HIV, can reactivate TB infection to TB disease [6]. In recent years, human migration patterns have contributed to the incidence of TB in industrialized countries. Hence, a combination of both sociodemographic factors and impaired immune response has led to persistence and dissemination of TB in locations where cases used to be sporadic.

Here we discuss a rare case of TB presenting as a gastric abscess in a patient with AIDS.

## 2. Case Presentation

A 49-year-old Hispanic female with AIDS, nonadherent to antiretroviral therapy (ART), with history of long-standing, mild (2/10), intermittent abdominal discomfort for over two years, presented to our hospital with worsening epigastric pain for four days and fever for two days. Epigastric pain was intermittent and squeezing and associated with nausea. The patient had experienced fatigue for one month prior to admission. Review of systems was negative for anorexia, weight loss, night sweats, shortness of breath, bowel, or urinary complaints. She denied prior tuberculosis exposure. Patient had been noncompliant with multiple ART regimens citing gastric intolerance. ART regimens included lopinavir, ritonavir, darunavir, emtricitabine, tenofovir, nevirapine, etravirine, and raltegravir. She frequently missed her appointments at the outpatient HIV clinic.

The patient was diagnosed with HIV infection in 1997, which is thought to be sexually acquired. Her last CD4 count was 21 cells/*μ*L (2%) with a viral load of 24,800 copies/mL, two months prior to admission. The patient was taking dapsone 100 mg daily for* Pneumocystis jirovecii* prophylaxis. She was born and raised in New York City and was unemployed. She denied toxic habits. She had traveled to Dominican Republic in 1997. Her family history was noncontributory.

**Figure 1:**
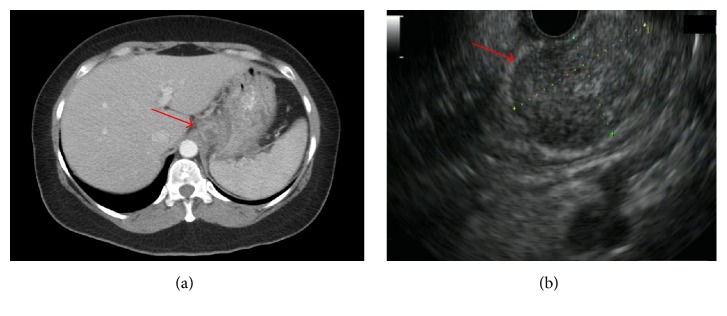
Computer tomography (CT) scan revealing a 1.2 × 1.0 cm low attenuation lesion adjacent to the gastric fundus ((a) red arrow); the lesion was also visualized on endoscopic ultrasound ((b) red arrow).

On admission, the patient appeared well nourished and febrile (39°C), with oropharyngeal thrush but no palpable lymphadenopathy. She had mild epigastric tenderness with no rebound or guarding. The rest of the examination was unremarkable. Laboratory work showed a white cell count of 7500/*μ*L (normal range: 4,800–10,800/*μ*L) with 75% neutrophils. Hematocrit was 34.3% (range 37–47%). Serum electrolytes, liver function tests, and urinalysis were normal. Toxoplasma serology was negative. A computed tomography of the abdomen with contrast revealed a 1.2 cm × 1.0 cm low attenuation lesion adjacent to the gastric fundus, suggestive of an abscess versus necrotic adenopathy (Figure 1(a)). The chest radiograph was unremarkable.

**Figure 2:**
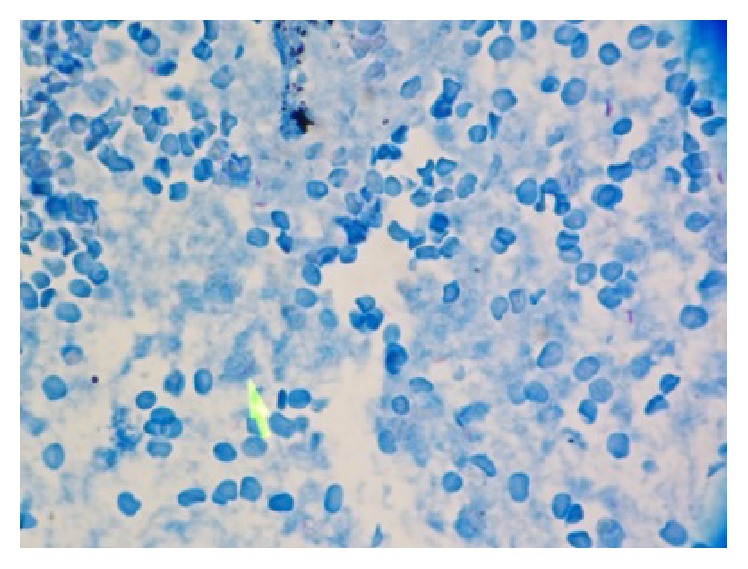
Acid-fast stain showing AFB positive bacteria (small red bacilli). Magnification 900x.

The patient was started on empiric intravenous vancomycin and piperacillin/tazobactam. Oral fluconazole was added for oral candidiasis. She was started on azithromycin 1200 mg weekly for MAC prophylaxis. Given low risk for nosocomial pathogens the antibiotics were narrowed to amoxicillin/clavulanate. She underwent esophagogastroduodenoscopy (EGD) which showed a normal esophagus. Atrophic gastric mucosa was seen in the body and enlarged folds with friable mucosa and lacy reticular pattern were seen in the gastric fundus. The duodenum appeared normal. Esophageal and gastric biopsies showed focal active gastritis in the fundus with chronic inactive gastritis and lymphoid hyperplasia in the body. No granuloma or acid-fast bacilli (AFB) were seen. Upper Endoscopic Ultrasound (EUS) revealed a 3.1 cm × 1.8 cm heterogeneous mass lesion arising from the submucosa in the fundus with small anechoic spaces (Figure 1(b)). Viscous serosanguinous liquid was obtained by fine needle aspiration. Gram stain of the aspirate was negative and AFB smear showed numerous organisms. Biopsy of the gastric lesion showed sheets of polymorphonuclear cells and few histiocytes consistent with an abscess. No granulomas or malignant cells were seen. Multiple AFB were seen by Kinyoun and Ziehl-Neelsen staining (Figure 2).

Patient's fevers resolved with improvement in abdominal symptoms within 72 hours after aspiration and antibiotics. QuantiFERON-TB Gold test was positive at 6.61 IU/mL. Serum cryptococcal antigen and urine* Histoplasma* antigen were negative. She was started on rifampin, isoniazid, pyrazinamide, ethambutol, and moxifloxacin to cover MTB,* M. bovis*, and* M. avium-intracellulare* complex (MAC). Bacterial, mycobacterial, and fungal blood cultures were negative. DNA polymerase chain reaction (PCR) for MTB was positive and DNA polymerase chain reaction (PCR) for MAC was negative. MTB grew from the gastric aspirate culture after 13 days. The isolate was pan-susceptible to antimycobacterial agents and ethambutol was discontinued. Stool AFB stain was positive and MTB was identified in the stool. Active pulmonary disease was ruled out by negative AFB stains in serial induced sputum and unremarkable chest imaging. The patient tolerated the medications and was discharged to follow-up in the clinic for initiation of ART.

She was started on emtricitabine, tenofovir, and raltegravir three weeks after discharge. Rifampin was switched to rifabutin to avoid drug interaction with raltegravir. Moxifloxacin was stopped after MAC infection was ruled out. The patient tolerated antitubercular therapy (ATT) and gained 20 pounds in four weeks. Her CD4 count improved to 72 cells/*μ*L (8%) and viral load dropped to 56 copies/mL in four months after initiation of ART.

## 3. Discussion

In general, TB mostly affects the lungs. However, primary MTB infection can occur at extrapulmonary sites, especially in individuals with impaired immunity such as HIV-infected patients. The gastrointestinal (GI) tract is the sixth most common site of extrapulmonary TB. Prior reports have shown that the confinement of GI TB to the stomach is unusual [7]. It is most commonly seen in the ileocecal region, followed by the ascending colon, jejunum, appendix, duodenum, stomach, esophagus, sigmoid colon, and rectum. Gastric TB is rare due to the lack of gastric mucosal lymphoid tissue, high acidity, rapid gastric transit, and local immunity of the stomach wall [8]. The pylorus is the site most commonly affected due to the higher number of lymphoid follicles [7, 9]. The fundus is less commonly affected. Gastric TB is usually secondary to other forms of TB infection, most often pulmonary TB. Purported routes of infection include lymphatic spread from adjacent celiac lymph nodes, hematogenous and less commonly direct mucosal invasion, extension from adjacent structures, and superinfection of an ulcer or malignancy. Our patient had no evidence of another primary source.

Gastric TB most commonly occurs as an ulcerating lesion with or without gastric outlet obstruction. Sometimes these lesions can lead to upper GI bleed, perforation, and rarely gastrobronchial and gastrocolic fistulae. The hypertrophic infiltrating form of gastric TB causes gastric or periampullary masses manifesting as pyloric obstruction mimicking gastric malignancy. Miliary involvement of the stomach is less common. Finding of an abscess is very unusual among gastric TB cases described in the literature. Only a few reports of submucosal lesions have been published where exudative material and granulomatous inflammation were found [10, 11]. Other cases have previously been reported as solid gastric masses without abscess formation and the diagnosis has been made based on microscopic tissue examination or PCR [12, 13]. About 10% of gastric TB cases present concurrently with gastric cancer [14]. In our case, the classic features of an abscess on imaging suggested an infectious etiology and not malignancy with pyogenic bacteria being the initial suspects.

Gastric abscess is a rare disorder and is mainly caused by bacteria in the oral cavity. Seventy to seventy-five percent of cases are caused by* Streptococcus* spp. [15, 16].* Escherichia coli*,* Proteus vulgaris*,* Proteus mirabilis*,* Haemophilus influenzae*,* Clostridium welchii*,* Pseudomonas aeruginosa*,* Staphylococcus*, and* Bacillus subtilis* have also been implicated [15, 16]. Of note, our patient had a history of prior gastric intolerance to ART and had a normal upper GI series a year prior to admission. The finding of sheets of PMNs on histopathology suggests that the patient could have had a superimposed bacterial infection over the ongoing tubercular process that might have worsened her chronic symptoms and led to her acute presentation. Bacteria, however, were not isolated from the aspirate likely due to use of empiric antibacterials before biopsy.

Surprisingly, the aspirate was loaded with MTB with no signs of granulomatous inflammation as described in prior case reports [10, 11]. This could be the result of advanced immunodeficiency represented by patient's low CD4 cell count. Absence of granulomatous changes has been found in tuberculous lymphoid tissue biopsies of patients with CD4 counts <50/*μ*L [17]. This finding has been related to the lack of expression of the IL-2 receptor (CD25) in lymphocytes of patients with advanced AIDS. The presence of CD25 has been involved in the transformation of macrophages into epithelioid cells, one of the hallmarks of granuloma formation [18].

Treatment of tuberculosis in AIDS warrants prompt initiation of ART along with antitubercular therapy [19]. Endoscopic aspiration of the gastric abscess with antibiotics and ATT along with ART led to clinical improvement in our patient.

## 4. Conclusions

High index of suspicion for gastric TB in HIV patients is necessary to avoid delay in diagnosis and to decrease morbidity and mortality. Unlike in the past, when gastric TB was mainly diagnosed at laparotomy or postmortem, advent of EUS-guided biopsy has made TB diagnosis faster and easier. This case illustrates yet another rare extrapulmonary presentation of tuberculosis in patients with AIDS.
